# Supplementary material for: What does it take to learn the rules of RNA base pairing? A lot less than you may think
Source: bioRxiv. 2025 Aug 2:2025.07.31.668042. Preprint. [Version 1] doi: 10.1101/2025.07.31.668042 (PMC12324431; doi:10.1101/2025.07.31.668042)
Supplement: Supplement 1 [file media-1.gz › supplemental_material/src/rscape_v2.6.0.tar.gz!/rscape_v2.6.0/lib/infernal/Manuscripts/filter_thresholds/figs/5S_sh.pdf]

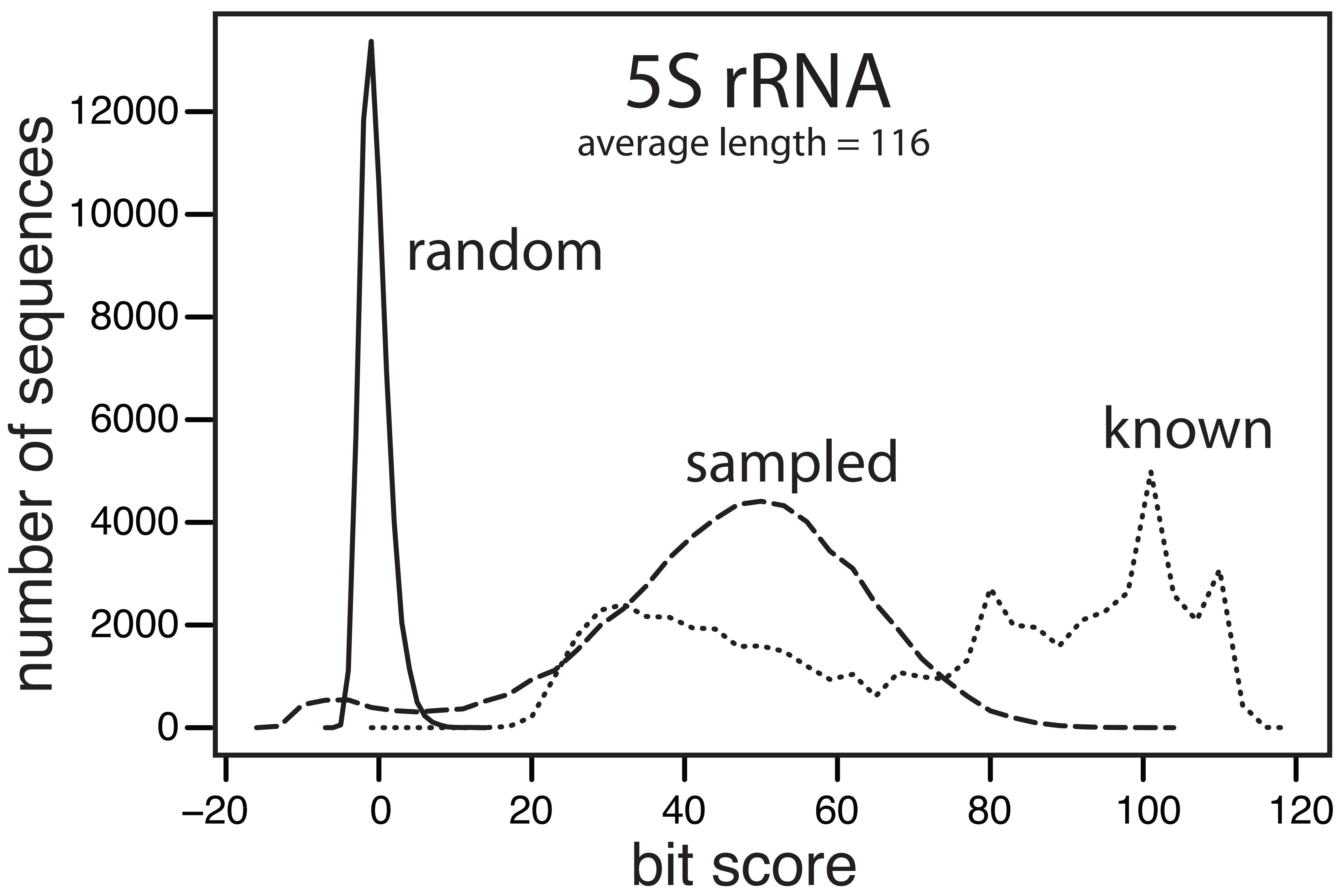
5S rRNA
average length = 116
random
sampled
known
number of sequences
0
2000
4000
6000
8000
10000
12000
bit score
–20
0
20
40
60
80
100
120
